# Perampanel inhibits calcitonin gene-related peptide release from rat brainstem in vitro

**DOI:** 10.1186/s10194-018-0940-5

**Published:** 2018-11-12

**Authors:** Giuseppe Tringali, Diego Currò, Pierluigi Navarra

**Affiliations:** Institute of Pharmacology, Fondazione Policlinico Universitario A. Gemelli IRCSS, Roma - Università Cattolica del Sacro Cuore, Largo Francesco Vito 1, 00168 Rome, Italy

**Keywords:** AMPA, Brainstem, Calcitonin gene-related peptide, Glutammatergic, Perampanel, Innovative biotechnologies, Rat

## Abstract

**Background:**

Perampanel is a novel antiepileptic drug acting via non-competitive antagonism on glutamatergic AMPA receptors, and the subsequent inhibition of ion calcium influx. Since it was recently postulated that the antagonists of glutamate receptors might play a role in the treatment of migraine, in this study we investigated the putative anti-migraine activity of perampanel in an in vitro animal model involving the static incubation of rat brainstem explants and the subsequent measurement of immune-reactive calcitonin gene-related peptide released into the incubation medium.

**Methods:**

Acute rat brainstem explants were incubated in plain medium or in medium containing graded concentrations of perampanel. The release into the medium was assessed by radioimmunoassay either under baseline conditions or after stimulation by such secretagogues as high K^+^ concentrations, veratridine or capsaicin.

**Results:**

We found that: 1) under baseline conditions perampanel, given in the range 0.01–100 μM, inhibited in a concentration-dependent manner calcitonin gene-related peptide’s release compared to controls; the decrease was statistically significant as from 10 μM; 2) a significant and consistent increase in calcitonin gene-related peptide’s secretion was induced by all depolarizing stimuli after 1 h of incubation; 3) under these conditions, calcitonin gene-related peptide’s release stimulated by 56 mM KCl was significantly reduced by perampanel from 0.1 μM onward, whereas secretion stimulated by veratridine was significantly reduced as from 1 μM; 4) on the contrary, perampanel had no effect on capsaicin-induced calcitonin gene-related peptide’s release up to 100 μM.

**Conclusions:**

Here we provided preliminary in vitro evidence suggesting that perampanel might control pain transmission under conditions of activated trigeminal system, in a preclinical model mimicking the pathophysiology of human migraine.

## Background

Perampanel is a new chemical entity, the first-in-class of antiepileptic drugs (AEDs) acting on the modulation of glutamatergic post-synaptic transmission via non-competitive AMPA antagonism [1]. Pre-clinical studies showed that perampanel inhibits AMPA-induced Ca^++^ influx in isolated rat cortical neurons in a concentration-dependent manner, with an IC50 of about 0.1 μM [2]. The binding of radiolabeled perampanel to neuronal rat membranes was not displaced by glutamate, AMPA or AMPA receptor antagonists given up to 1 mM, whereas non-competitive AMPA antagonists such as CP465022 or GYK152466 effectively displaced perampanel [2]; such drug-receptor interaction is fully consistent with the functional effects exerted by perampanel on Ca^++^ influx. Perampanel has been approved for the treatment of epileptic patients; the drug is currently indicated for the adjunctive treatment of partial-onset seizures as well as primary generalized tonic-clonic seizures, both in adults and pediatric patients from 12 years on. The clinical efficacy of perampanel in partial-onset seizures was demonstrated in 3 double-blind, randomized placebo-controlled Phase-III trials (studies 304, 305 and 306) [3–5], followed on a long-term extension trial (study 307) [6]. Later on, the efficacy in the treatment of primary generalized tonic-clonic seizures was shown in a further randomized controlled trial, study 332 [7]. In all studies, perampanel or placebo were given on top of standard-of-care AEDs therapies.

Based on the existence of common patho-physiological features linking epilepsy to migraine [8], it has been recently postulated that the antagonists of glutamate receptors may play a role in the treatment of migraine [9]. In fact, glutamate receptors have been localized in areas related to migraine patho-physiology, including the trigeminal ganglion, trigeminal nucleus caudalis and thalamus [10, 11], where their stimulation by glutamate activates neurons in the trigeminal nucleus caudalis [12]. Moreover, various kainate and glutamate receptor antagonists proved effective in animal models of migraine (reviewed in [9]), which encouraged a number of pilot clinical studies with mGluR5, AMPA and/or kainate receptor antagonists in acute migraine [13–15].

Within the framework of AEDs and migraine, in the present study we tested the hypothesis that perampanel can modulate the release of immune-reactive calcitonin gene-related peptide (CGRP, a peptide neurotransmitter most important in migraine pathophysiology [16]), from acute rat brainstem explants. To this purpose, we used a previously validated in vitro model, which proved useful in studies investigating the effects of various agents – notably including AEDs - on the synaptic junctions between primary and secondary neurons along the pain neurotransmission pathways [17–19].

## Methods

### Chemicals

Perampanel [2-(2-oxo-1-phenyl-5-pyridin-2yl-1,2-dihydropyridin-3-yl) benzonitrile] was a kind gift by Eisai Co., Ltd. (Tokyo, Japan). Batch 173H2901 was used for this set of experiments. Perampanel powder was stored at 4 °C. All solutions were freshly prepared before use. The drug was dissolved in DMSO solution to obtain 10 mM stock solutions; further dilutions were made in the incubation medium or in a medium consisting of 56 mM KCl (see section: “Brainstem incubations”).

Veratridine and capsaicin were purchased from Sigma (Sigma Chemicals Co., St. Louis, MO, USA), and dissolved in 100% ethanol at 10 mM concentration; subsequently, the standard solutions were diluted with incubation medium to reach the desired final concentrations.

Neither DMSO nor ethanol interfered with CGRP release when used at working concentrations (i.e 0.1% or less). Furthermore, none of the test drugs used interfered with CGRP assay.

### Animals

Male Wistar rat aged 8–12 weeks (weight range 220 – 275 g) were used. Animals, obtained from the Animal Facility of Catholic University, were housed under a 12-h light-dark cycle at room temperature with free access to food and drinking water; body weight was weekly monitored. All animal procedures were approved by the Italian Ministry of Health (licensed authorization to P. Navarra n.648/2017-PR) and were carried out in such a way as to minimize the suffering of the animals and the number of animals used.

### Brainstem incubations

The entire experimental procedure has been previously described in detail [19, 20]. In brief, on the day of experiment, the animals were decapitated and the brains rapidly removed. After removal of the cerebellum, the brainstems were dissected within their anatomical limits and subsequently incubated in a 24-well plates (one brainstem per well) in 500 μl of incubation medium [Minimum Essential Medium with Earle’s salts (MEM), supplemented with bovine serum albumin, glutamine, ascorbic acid and aprotinin; pH 7,4] at 37 °C in a humidified atmosphere consisting of 5% CO2 and 95% O2. Under these conditions, brainstem explants remained viable and functional during the timeframe of the experiments and variations in CGRP release did not appeared to be correlated with toxic damage of the tissues.

**Fig. 1:**
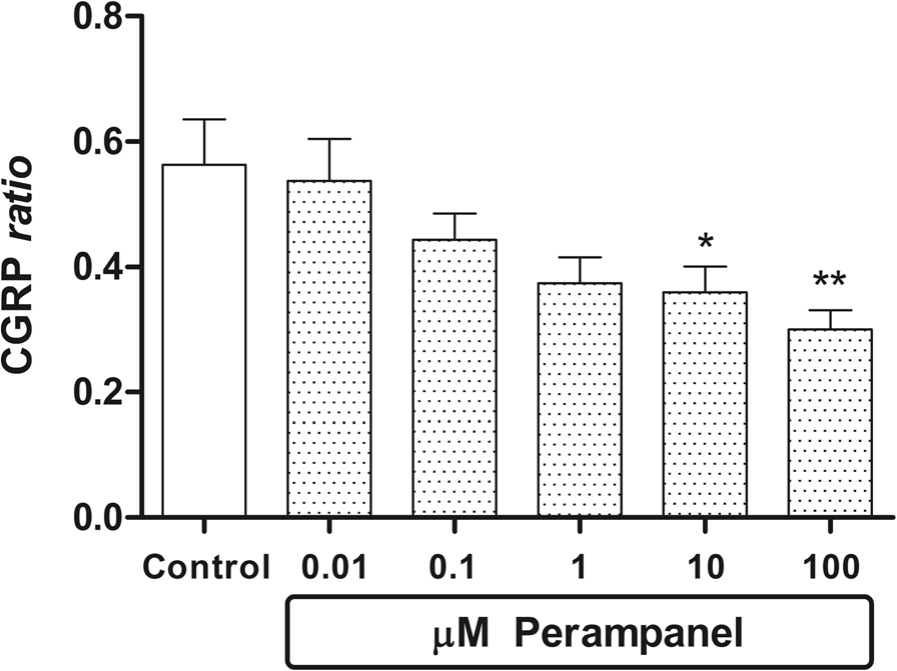
Inhibitory effect of perampanel on basal CGRP release from rat brainstem explants. Data are expressed as CGRP ratio (see “Materials & Methods” section), the means ±1 S.E.M. of 9 replicates per group. In the figure, asterisks refer to statistical comparisons of each experimental group versus unstimulated controls. In particular, * and **: *p* < 0.05 and *p* < 0.01 vs Control respectively

**Fig. 2:**
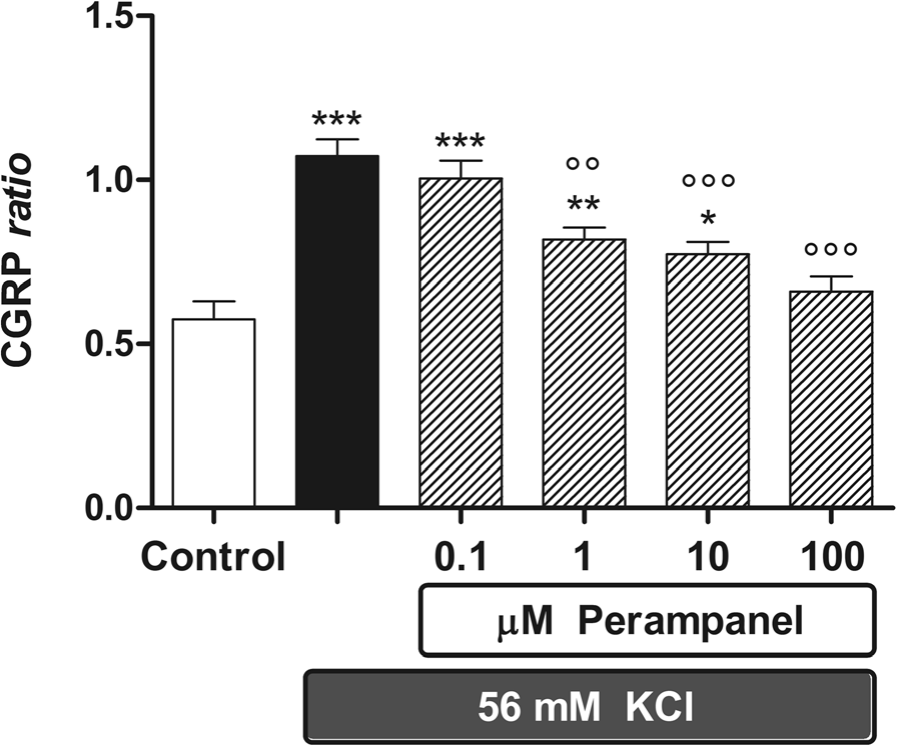
Perampanel reduces in significant manner CGRP release stimulated by 56 mM KCl from rat brainstem explants. Data are expressed as CGRP ratio (see “Materials & Methods” section), the means ±1 S.E.M. of 9 replicates per group. In the figure, asterisks refer to statistical comparisons of each experimental group versus unstimulated controls, whereas circles refer to statistical comparisons of each experimental group versus secretagogue-stimulated controls. In particular, *, ** and ***: *p* < 0.05, *p* < 0.01 and *p* < 0.001 vs Control respectively; °° and °°°: *p* < 0.01 and *p* < 0.001 vs the secretagogue given alone, respectively

**Fig. 3:**
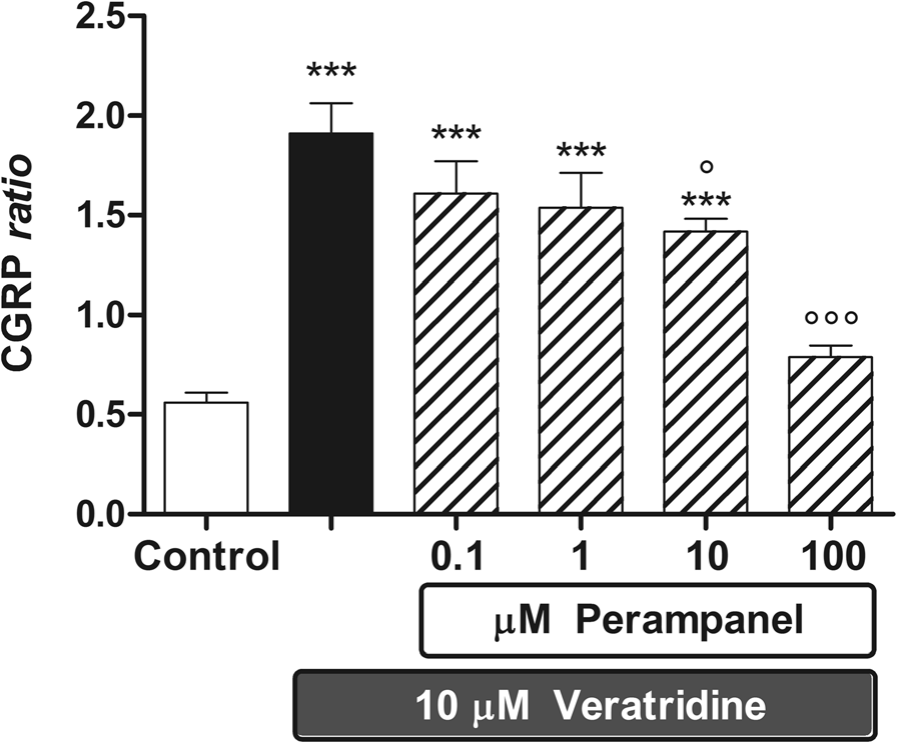
The effect of perampanel on veratridine-stimulated CGRP release from rat brainstem explants. Data are expressed as CGRP ratio (see “Materials & Methods” section), means ±1 S.E.M. of 10 replicates per group. In the figure, asterisks refer to statistical comparisons of each experimental group versus unstimulated controls, whereas circles refer to statistical comparisons of each experimental group versus secretagogue-stimulated controls. In particular, ***: *p* < 0.001 vs controls; ° and °°°: *p* < 0.05 and *p* < 0.001 vs veratridine given alone, respectively

**Fig. 4:**
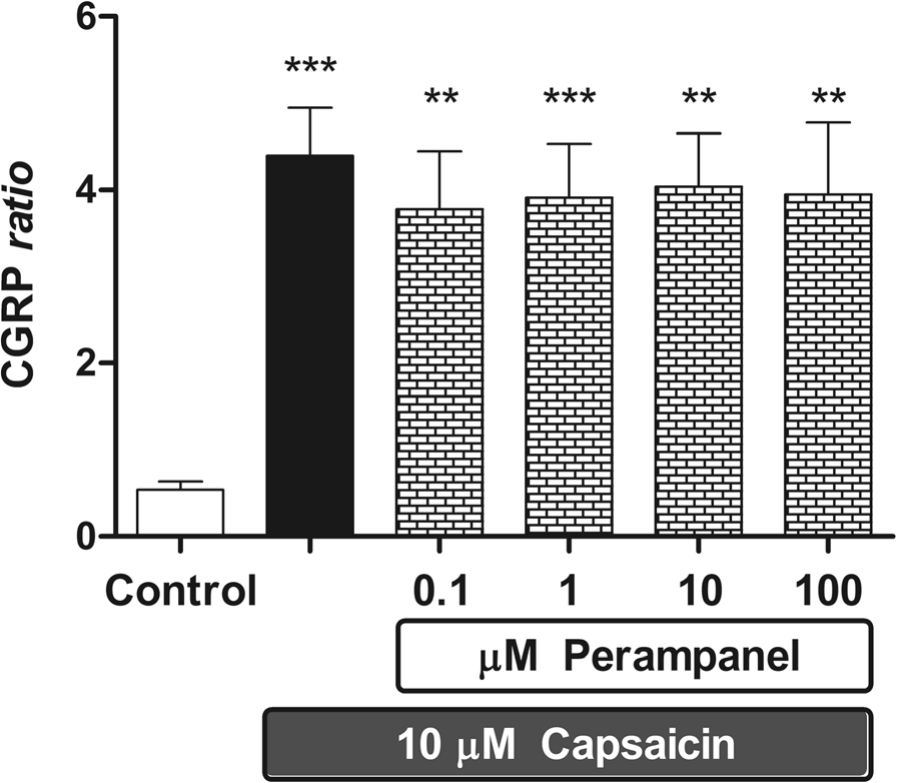
The effects of perampanel on capsaicin-stimulated CGRP release from rat brainstem explants. Data are expressed as CGRP ratio (see “Materials & Methods” section), the means ±1 S.E.M. of 7 replicates per group. In the figure, asterisks refer to statistical comparisons of each experimental group versus unstimulated controls. In particular,** and ***: *p* < 0.01 and *p* < 0.001 vs Controls, respectively

After 1 h pre-incubation (during which the medium was changed every 20 min), the explants were subjected to a 1-h incubation in medium alone, to assess basal CGRP release. In the second 1-h incubation medium, test substances were added to the medium. In particular: a) in experiments shown in Fig. 1, media contained graded concentrations of perampanel or medium alone in the control group; b) in experiments shown in Figs. 2, 3 and 4, media contained the secretagougue given alone, or the secretagogue additioned with graded concentrations of perampanel; in these experiments, one group with medium alone was taken to assess release under basal conditions. Whenever KCl was used, MEM was replaced by a medium consisting of 56 mM KCl and 67 mM NaCl, with the same concentration of the other ions as found in MEM. At the end of the first and second incubations, the media were collected and stored at − 35 °C until assay for CGRP immunoreactivity.

### CGRP radioimmunoassay

CGRP released in the incubation medium was measured by a radioimmunoassay technique developed and validated in our laboratory as previously described in detail [21].

### Statistical analysis

All data are expressed as CGRP ratio, obtained dividing the amount of CGRP released in the second 60-min incubation period by the amount released in the previous 60 min period (paired controls). Expression of data as ratio minimizes CGRP variations among different tissue explants.

Each experiment was repeated three times (unless otherwise stated) according to a randomized block design [22]. Thereafter, data were analysed by one-way ANOVA and subsequent post-hoc Newman–Keuls for comparisons between group means, using a PrismTM computer program (GraphPad, San Diego, CA, USA). *P* values lower than 0.05 (*p* < 0.05) were considered significant.

## Results

Under baseline conditions perampanel, given in the range of concentrations 0.01–100 μM, inhibited in a concentration-dependent fashion CGRP release compared to controls treated with vehicle; the decrease was statistically significant from 10 μM onward (Fig. 1). Results shown in this figure are obtained pooling three independent experiments, each performed in triplicate, to a total of 9 replicates per experimental group. Maximal percent inhibition versus basal release (− 46%) was achieved with 100 μM perampanel. Therefore, the estimated EC50 in this model is half of this maximal effect, i.e. -23%. The EC50 was achieved at a 0.1 μM perampanel concentration.

A significant and consistent increase in CGRP secretion was induced by specific (capsaicin) or nonspecific (56 mM KCl or veratridine) depolarizing stimuli after 1 h of incubation (Figs. 2, 3 and 4). Under these conditions, perampanel was able to antagonize the increase in CGRP secretion elicited by the two nonspecific secretagogues. In particular, secretion stimulated by 56 mM KCl was significantly reduced by perampanel as from 0.1 μM (Fig. 2), whereas secretion stimulated by veratridine was significantly reduced from 1 μM onward (Fig. 3). Results shown in Figs. 2 and 3 have been obtained pooling three independent experiments, each including 3 or 4 replicates per experimental group respectively, to a total of 9 replicates per experimental group in KCl experiments, and 10 replicates in veratridine experiments. Maximal percent inhibitions over KCl- and veratridine-stimulated CGRP release (− 39.5% and − 60%, respectively) was achieved by perampanel at 100 μM.

At variance, perampanel had no effect on capsaicin-induced CGRP release up to 100 μM (Fig. 4). Results shown in this figure are obtained pooling two independent experiments, performed in triplicate and quadruplicate respectively, to a total of 7 replicates per experimental group.

## Discussion

In this work, we found that perampanel is able to inhibit in a concentration-dependent manner basal CGRP release from isolated rat brainstem; the issue of CGRP released in the incubation medium as a marker of trigeminal activation has been discussed elsewhere [19]. Under these conditions, the size effect of perampanel over peptide release is − 46% compared to baseline release, obtained at a 100 μM perampanel concentration. Half of the size effect (i.e. -23%, representing an estimate EC50 in this model) is achieved at a 0.1 μM concentration, which corresponds to the EC50 of perampanel in reducing Ca^++^ influx in rat cortical neurons [2].

Likewise, perampanel was able to inhibit the release of CGRP stimulated by high KCl concentration or veratridine, but failed to antagonize the stimulatory effects of 10 μM capsaicin. The EC50 of perampanel on KCl-stimulated release was in the same order of magnitude of that observed under baseline conditions, whereas 1–10 μM of perampanel were needed to achieve the EC50 after veratridine stimulation. Probably such difference is related to the different mechanisms of action of the two secretagogues, since KCl solutions elicit direct Ca^++^ influx within the neurons (and the effect of perampanel is mostly based on Ca^++^ influx modulation), whereas veratridine acts primarily via Na + channel opening, although both Ca^++^ and Na^+^ channel activation is required [23]. As far as capsaicin is concerned, this is a specific, receptor-operated stimulus, and it is a far more effective secretagogue in this experimental model (in this study, 8.5-fold increase versus basal release, compared to 3.5-fold and 2-fold increases elicited by veratridine and KCl, respectively). In our experience, other agents -notably including morphine and lacosamide- failed to counteract capsaicin, whereas tapentadol and reboxetine had a weak inhibitory effect in the millimolar range [18, 19]. We have previously interpreted these findings by concluding that the effect of capsaicin is too strong, and the same reasoning might apply to perampanel as well.

Here we report preclinical in vitro evidence that perampanel might be useful in the treatment of disorders related to inappropriate CGRP secretion, whose primary clinical presentation in humans is migraine. There is also evidence that perampanel was found effective in a model of neuropathic pain in the rat [24]. Taken together, these findings represent an initial background suggesting the opportunity to test perampanel in the clinical setting of migraine. At this stage, some additional pre-clinical data (such as, for instance, recordings of neuronal activity in the trigeminal nucleus caudalis of animal models) might further encourage researchers of the field toward clinical investigations. Indeed, other investigational agents related to perampanel (including the AMPA antagonist BGG492, the kainate antagonist LY466195, the AMPA/kainate antagonist tezampanel and the mGluR5 antagonist ADX10059) have been tested in proof-of-concept trials involving up to 128 patients, starting from comparable nonclinical bases (reviewed in [9]). Compared to the above-mentioned investigational tools, perampanel may present some advantage regarding clinical studies. First, perampanel is already approved in two different populations of epileptic patients, and the profile of safety emerging from clinical studies on a larger set of patients, along with data coming from post-marketing surveillance, is far better defined compared to those of other glutamate antagonists at earlier phases of clinical development. Second, a large array of strengths is available, making easier the design and conductance of dose-finding studies. Moreover, perampanel presents a favorable pharmacokinetic profile; after oral administration, the drug is almost completely absorbed, and Cmax plasma levels are reached within 0.5–2.5 h [25], which fits well with the oral administration at the onset of a migraine attack.

The therapeutic area of migraine treatment has recently undergone a fast growth, because of the arrival of novel anti-CGRP antibodies [26]. While these innovative drugs are highly effective and well tolerated, they all are approved for migraine prevention, meaning that the frequency of migraine attacks in patients treated with anti-CGRP antibodies is significantly reduced but not abolished [26]. Thus, the effective treatment of migraine attacks remains a partially unmet need, which warrants studies on novel therapeutic options.

## Conclusions

Here we showed that the novel antiepileptic agent perampanel is able to inhibit both basal and secretagogue-stimulated CGRP release from isolated rat brainstems; the latter is a validated preclinical model that our group has largely used in the past to investigate the role of potential anti-nociceptive agents in pain neurotransmission. We have also discussed the issue of testing the efficacy of perampanel in the clinical setting of migraine, providing some argument in support of this opportunity.

## Abbrevations

AEDs: Antiepileptic drugs
AMPA: α-amino-3-hydroxy-5-methyl-4-isoxazolepropionic acid
ANOVA: Analysis of variance
CGRP: Calcitonin gene-related peptide
DMSO: Dimethyl sulfoxide
KCl: Potassium chloride
MEM: minimum essential medium
NaCl: Sodium chloride
